# New Test for Arsenic

**Published:** 1871-02

**Authors:** 


New Test for Arsenic.—Bettendorf has found a test so delicate that one part of arsenic in 1,000,000 parts of solution may be detected, and the presence of antimony does not affect it. To apply this test, the suspected liquid is mixed with hydrochloric acid until fumes are apparent. Chloride of tin is then added, and a basic precipitate containing the greater part of the arsenic as a metal, mixed with the oxyde of tin is thrown down.—*Cincinnati Med. Reporter.*

